# Demonstration of a laparoscopic structured-illumination three-dimensional imaging system for guiding reconstructive bowel anastomosis

**DOI:** 10.1117/1.JBO.23.5.056009

**Published:** 2018-05-23

**Authors:** Hanh N. D. Le, Hieu Nguyen, Zhaoyang Wang, Justin Opfermann, Simon Leonard, Axel Krieger, Jin U. Kang

**Affiliations:** a Johns Hopkins University, Department of Electrical and Computer Engineering, Baltimore, Maryland, United States; b Catholic University of America, Department of Mechanical Engineering, Washington, DC, United States; c Children’s National Health System, Sheikh Zayed Institute for Pediatric Surgical Innovation, Washington, DC, United States; d Johns Hopkins University, Department of Computer Science, Baltimore, Maryland, United States; e University of Maryland, Department of Mechanical Engineering, College Park, Maryland, United States

**Keywords:** endoscopic imaging, three-dimensional image acquisition, anastomosis, structured illumination

## Abstract

This paper reports the development and system analysis of a laparoscopic system based on structured illumination technique capable of three-dimensional (3-D) reconstruction of porcine intestine during surgical anastomosis (connection of tubular structures). A calibration target is used to validate the system performance and results show a depth of field of 20 mm with an accuracy of 0.008 mm and precision of 0.25 mm. The imaging system is used to reconstruct a quantitative 3-D depth measurement of *ex vivo* porcine bowel tissues to mimic an end-to-end bowel anastomosis scenario. We demonstrate that the system can detect a suture in the tissue and map homogeneous surfaces of the intestine with different tissue pigments, affirming the feasibility for depth quantization for guiding and assisting medical diagnostic decisions in anastomosis surgery.

## 1 Introduction

Modern surgical procedures have increasingly adopted minimally invasive image-guidance surgical techniques to reduce surgical trauma. A typical minimally invasive surgical guidance system provides visual assistance of the surgical scene qualitatively in two and recently also in three dimensions within a limited field of view (FOV). The small FOV and narrow peripheral vision inherent to current endoscopic technology create highly variable perceptions of the surgical field, which impacts each surgeon’s hand–eye coordination, learning curve, performance, and clinical outcome.^1^^–^^3^ These human variations can critically affect functional outcomes in all complex surgeries such as cardiac valvular suturing, where precision and accuracy of suture placement on a millimeter or two scale can make a significant difference.^4^^–^^7^ For surgical tasks such as anastomosis, quantitative three-dimensional (3-D) measurement would benefit both surgeon and surgical robots. This technology enables more precise depth perception than by two-dimensional (2-D) imaging, by enhancing the depth information a user perceives.^8^ Variations in 3-D reconstruction techniques for quantitative imaging include stereoscopy, time of flight, plenoptic, and structure illumination and have reported depth discrimination in endoscopy.

Stereoscopy relies on searching for stereo correspondences between two distinct but overlapped views of the object to produce a disparity map for 3-D reconstruction. Advances in disparity searching have improved the technique to be more cost-effective in both spatial and temporal dimensions^9^^,^^10^ and with high resolution up to 50  μm using optimal feature-based reconstructions.^11^^,^^12^

Time-of-flight (ToF) camera calculates the time difference between the emitted and reflected light to the sensor for 3-D reconstruction. Because ToF does not rely on correspondence search or baseline restriction, a ToF system can be compact; however, the technique depends strongly on the quality of the return rays, and sensor temperature tolerance.^13^ Depth resolution using 3-D ToF surgical endoscope varies largely from 0.89^14^ to 4 mm.^15^

Plenoptic imaging or light field imaging is a derivation of stereoscopy, in which more than two distinct and overlapped micro images are used for disparity searching. These microimages are formed at once on the sensor through multiple reflected rays from the object through a microlens array located in front of a camera sensor. With higher dimension of searching and the predefined microlenses geometry, plenoptic imaging is faster and compact. A demonstration of applying plenoptic in endoscopic setting was recently shown to be within 1 to 2 mm.^16^^,^^17^

Structured illumination (SI) provides depth quantification based on the parallax and triangulation of object location to the camera and the projector. For *in vitro* medical application, SI has been used widely in dermatology for skin and wound inspection^18^^–^^20^ and health-care for idiopathic scoliosis diagnostic.^21^ However, SI endoscopic imaging for medical intraoperative inspection has been so far rather limited. Due to limited working volume, many SI endoscopic designs do not consider the baseline between projector and the camera but use a common beam-splitter between them, resulting in nonuniform depth accuracy and restricted range of 3-D measurement,^22^ or providing only perceptual depth measurement.^23^^,^^24^ Most efforts applying SI in confined spaces, such as fiber or endoscope,^25^^–^^27^ have been reported with static and nontissue objects.^28^ Some highlighted studies of 3-D reconstruction on tissue use high-power light grids with unique spectrum and triangulate the light-of-sight rays from the light source to the camera on tissue features^29^^–^^31^ with submillimeter accuracy.

In this paper, we propose an imaging system for 3-D reconstruction of biological tissue in an endoscopic approach. We contribute a unique gradient gray-coded pattern for 3-D tissue reconstruction, where the discontinuity of the object is solved by phase calculation from a sequence of low- to high-frequency grid patterns to minimize the cost of color-coded illumination. The coded pattern features the shape of the tissue in vertical grids, deducing object’s geometry across its entire surface, instead of feature based as in stereoscopy, hence, reducing data “holes” in the reconstructed point cloud. Moreover, the coded patterns are generated using low-power light-emitting diode (LED), reducing the speckle pattern occurrence or specular reflectance in high coherence light source as often used in SI endoscopic imaging using supercontinuum or laser source. In addition, the baseline between camera and projector is utilized to provide high quantitative depth measurement. A model of *ex vivo* porcine intestinal tissue, which is similar to the preclinical anastomosis setting, is used to reconstruct the 3-D geometry of the surgical scene with the proposed endoscope. We show that the system can detect the tissue features, the location of the suture line, as well as the deformity of the tissue caused by certain tool manipulations.

## 2 Material and Method

### 2.1 System Setup

**Fig. 1:**
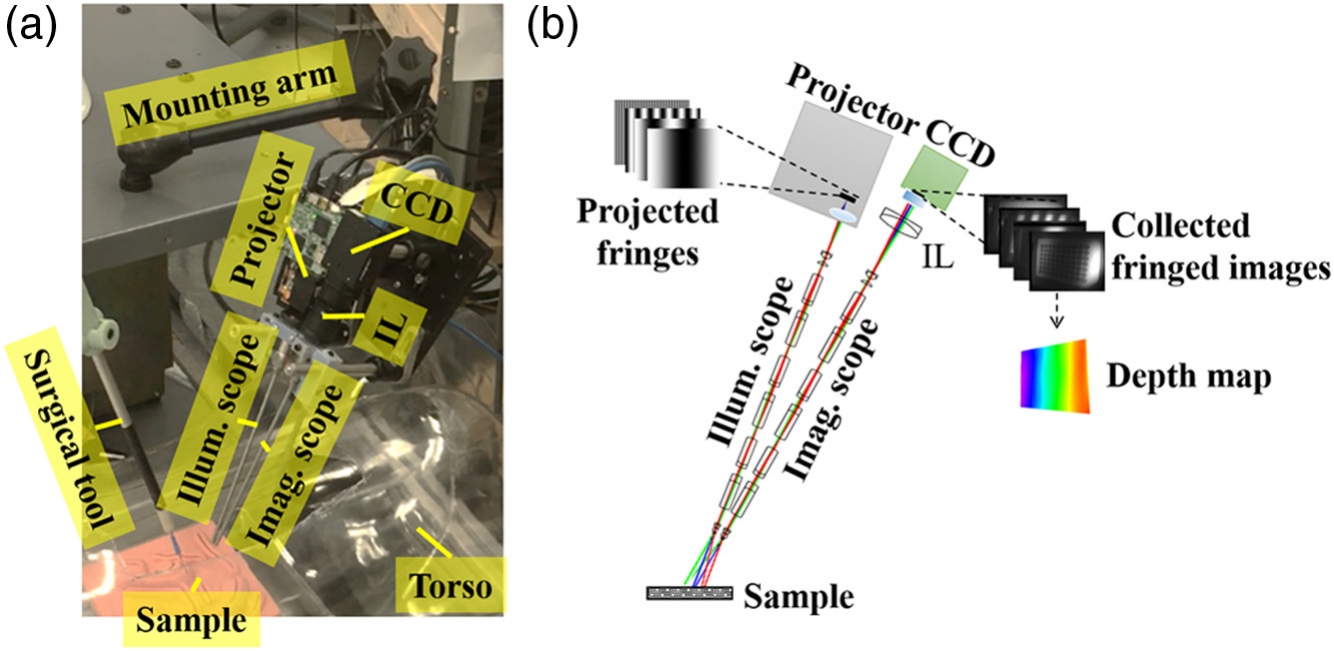
(a) Experimental setup of the 3-D endoscopic imaging system on a mounting arm with the surgical tool on the other arm (not shown) when targeting sample inside a torso phantom. (b) The schematic layout of the 3-D endoscope with simulated relay optics inside the rigid scopes. An example of 3-D reconstruction inputs is demonstrated with several vertical fringes loaded to the projector. These projections are delivered through the illum. scope optics, reflected off the sample surface and collected to the CCD through the imag. scope and IL optics, producing collected fringed images for depth map result.

To optically monitor how the tissue is deformed, we developed a 3-D endoscope based on SI technique (Fig. 1). The system consists of two identical rigid surgical endoscopes (HOPKINS 7230AA, 0 deg, 4-mm-diameter endoscope, Karl Storz GmbH & Co. KG, Tuttlingen, Germany) for illumination and imaging. The illumination endoscope (illum. scope) guides the modulated patterns projected by a digital micromirror device (DMD) (TI-DLP EVM3000, Texas Instrument, Dallas, Texas). These patterns shine on the objects, and their reflection off the sample is captured via the imaging endoscope (imag. scope), an achromatic doublet lens served as imaging lens (IL, AC254-060-A, Thorlabs, Newton, New Jersey) and a CCD camera (GS3-U3-15S5M-C, Point Grey Research Inc., Richmond, Canada). The projector is equipped with an optical chamber comprised of red, green, and blue LEDs. To make the current system adequate for imaging-pigmented sample, such as intestine, we use green LED for illumination as the camera responsivity is highest compared with other regions. The gray code is mentioned to indicate the dark gradient fringe follows a sinusoidal form [Eq. (1)]. For a low-noise triangulation and to minimize the overall form-factor of the endoscope, a minimum angle of 12 deg between the illumination and the imaging scopes is chosen to maintain the desired field of view and height accuracy.^32^^,^^33^ For the ray-tracing demonstration purpose, we adapted a standard endoscope model with the relay lens system based on a combination of rod lenses.^34^ The control interface is written in C# using multithread computation on a Dell Precision T7600 workstation and the optical system is modeled using Zemax Optics Studio 15 SP1 (Zemax, Kirkland, Washington).

### 2.2 Depth Reconstruction

3-D reconstruction using SI method is based on parallax and triangulation between the camera and the structured light from the projector, in relation to the sample surface profile. The technique involves linking an image point on the camera sensor to its corresponding point on the DMD of the projector via the collected fringe patterns. This is equivalent to treating the DMD as a second camera, similar to the stereovision technique. A representative reconstruction method is reported in the literature,^35^ where a governing equation was derived to relate the object depth information to the phase map of the projection fringes. In general, a vertical, phase-shifted sinusoidal fringe wave is used to perform the SI to achieve full field and fast processing. The fringe wave pattern is given as

$$I_{i}(u,v)=I_{o}[1+\cos(\frac{2\pi ku}{w}+\delta )],$$ (1)

where $I_{o}$ is the intensity modulation amplitude, (u,v) are the spatial pixel indices, δ is the shifted phase, k is the fringe number, and w is the pattern width. In our application, k={1,4,12,36}. This is defined uniquely for 3-D imaging of tissue sample such as intestines, providing us a smooth reconstruction without data “holes.”

After projection of the patterns and collection of the images, the wrapped phase of each obtained image with different fringe patterns and frequencies is calculated using the conventional four-step phase shift method as given as

$$\tan[\phi^{w}(u,v)]=\frac{I_{4}(u,v)-I_{2}(u,v)}{I_{1}(u,v)-I_{3}(u,v)},$$ (2)

where $I_{1-4}$ represents the intensity of shifted image, and w indicates wrapped phase, respectively.

In as much as both the original and the collected images have periodic structured patterns, the phase map at each point is restrained to a principal range, leading to the phase discontinuity in the case of high-frequency fringes. Therefore, a phase-unwrapping process is necessary to extract the absolute phase values. Our phase unwrapping algorithm is formulated according to the relation between the current and previous unwrapped phase information from the previous frequency. To facilitate the process, the lowest frequency patterns is defined to have a single fringe in it, which makes its unwrapped phase identical to the wrapped one.^36^ For other patterns of higher frequencies, the unwrapped phase distribution can be calculated from the unwrapped phase distribution of the previous frequency as follows:

$$\phi_{1}^{uw}(u,v)=\phi_{1}^{w}(u,v),$$ (3)

$$\phi_{n}^{uw}(u,v)=\phi_{n}^{w}(u,v)+2\pi \langle \frac{\phi_{n-1}^{uw}\frac{f_{n}}{f_{n-1}}-\phi_{n}^{w}}{2\pi }\rangle ,$$ (4)

where the operator ⟨•⟩ denotes the argument rounding to the closest integer, the superscript uw refers to unwrapped phase, f represents fringe frequency, i.e., number of fringes in the projection pattern, and n={2,3,4} is the n’th frequency of the fringes.

The out-of-plane depth z at each pixel index (i,j) is a function of the unwrapped phase, and it is expressed as

$$z=\frac{1+c_{1}\phi +(c_{2}+c_{3}\phi )u+(c_{4}+c_{5}\phi )v+(c_{6}+c_{7}\phi )u^{2}+(c_{8}+c_{9}\phi )v^{2}+(c_{10}+c_{11}\phi )uv}{d_{o}+d_{1}\phi +(d_{2}+d_{3}\phi )u+(d_{4}+d_{5}\phi )v+(d_{6}+d_{7}\phi )u^{2}+(d_{8}+d_{9}\phi )v^{2}+(d_{10}+d_{11}\phi )uv},$$ (5)

where $c_{1-11}$, $d_{o-11}$ are the constants determined by geometrical and other relevant parameters, ϕ is the unwrapped phase distribution. The second-order terms of u and v are adopted for the purpose of accuracy enhancement in practice.

The calibration to determine $c_{1-11}$ and $d_{o-11}$ can be performed using a flat calibration board with chessboard or circular patterns.^37^^,^^38^ The calibration board is translated and rotated at a number of positions and tilting angles to cover the imaging volume of interest. Each calibration control point j at a board position i on the calibration board is transformed to the corresponding point $\left(X_{c,ij},Y_{c,ij},Z_{c,ij}\right)$ in the camera coordinate system. The first board position (i=1) can be chosen as the reference plane, where the height or depth of the board surface is zero, and the board is usually placed nearly perpendicular to the optical axis of the camera. The description of the reference plane in the world coordinate system is formulated by fitting a planar equation with constant coefficients A, B, and C to the points in the first calibration board image

$$z_{ij}=\frac{AX_{c,ij}+BY_{c,ij}+CZ_{c,ij}+1}{A^{2}+B^{2}+C^{2}}.$$ (6)

After the calculation of the unwrapped phase ϕ and the depth $z_{ij}$ of each calibration control point j, the Levenberg–Marquard least-squares fitting algorithm is employed to obtain the coefficients $c_{1-11}$ and $d_{o-11}$ in Eq. (5) by minimizing the following cost function:

$$C=\overset{a}{\underset{i=1}{\sum }}\overset{b}{\underset{j=1}{\sum }}{\left(z-z_{ij}\right)}^{2},$$ (7)

where a is the number of board positions used for the calibration, and b is the number of control points on the calibration board.

### 2.3 System Validation

**Fig. 2:**
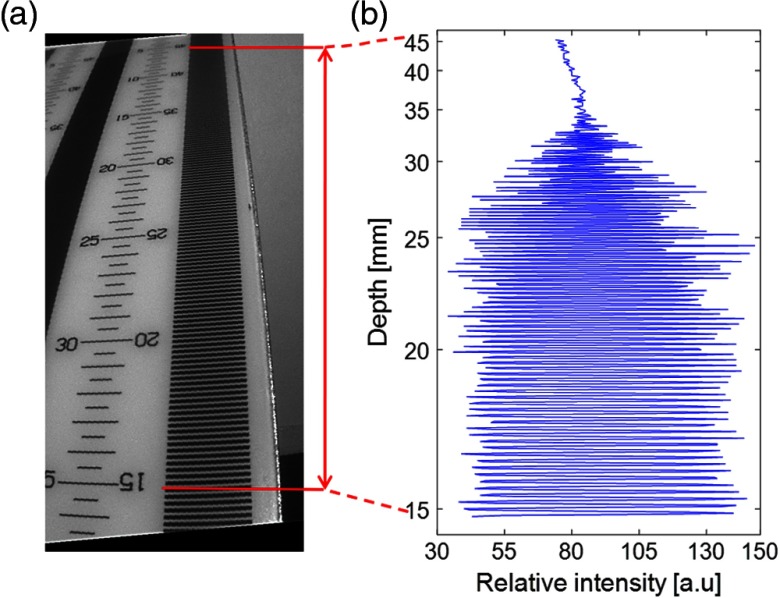
(a) Bright-field reflectance image of the depth of field target with red markers denoting the depth region for intensity profile plotting in Fig. 2(b) from marker 15 mm to marker 45 mm on the target surface. (b) The relative intensity profile along the selected depth segment.

To validate the performance of the system in terms of depth of field, field of view, and resolution, we used a depth of field target (DOF 5-15. Edmund Optics, York, UK) with a 45 deg inclination surface and line-pair ruler on its surface marking height variation. To minimize the specular reflectance from the projector light on the DOF target, the DOF was moved 10 mm away from the distal end of the endoscopes. The DOF is defined by the profile range in which the intensity contrast along a horizontal line-pair ruler on the DOF target is distinct. This DOF is measured by determining the region, where intensity contrast within the red segment [Fig. 2(a)] is the most distinct compared with other regions. The intensity of the ruler marker is shown in Fig. 2(b) from mark 15 to mark 45 mm on DOF depth ruler, indicating the highest contrast occurs between 15 and 35 mm, hence, object within 20 mm in height gives the best height accuracy. The field of view is varied depending on the distance between the endoscope’s distal end to the object, and the overlapped regions between the illumination and the imaging endoscopes, with endoscopic acceptance angle of 70 deg. In our setup, all the projected fringes are aligned within the imaging width, and the distal ends of the two scopes are angled at 12 deg.

**Fig. 3:**
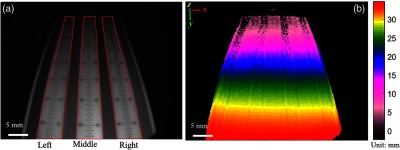
(a) Depth of field target with chosen left, middle, and right regions of interest for 3-D error assessment in Fig. 4. (b) The corresponding depth map of the DOF surface with depth color bar in millimeters. Camera exposure is set at 20 ms for each fringed image for the depth recording. The depth colormap denotes height measurements from the reference zero plane (farthest from the distal end of the scope) to the closest plane from the distal end of the scope.

**Fig. 4:**
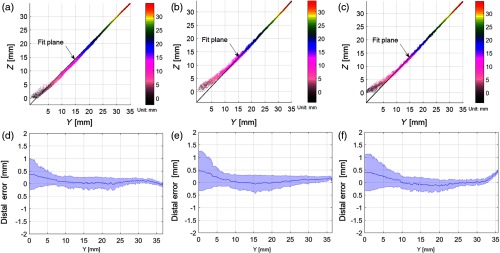
(a–c) 3-D point cloud population of left, middle, and right regions (marked as red ROIs from Fig. 3(a) with height coded color from the displayed color bar in millimeter). The plots are viewed along 45-deg angle plane of the DOF front surface. The displayed fitted plane is the plane calculated best fit of the point cloud population. (d–f) Precision of the depth measurement calculated in the left, middle, and right regions with blue middle lines indicating distal mean between 3-D point clouds to the fit plane, and the blue shade region indicating the standard deviation of the mentioned distal variations.

**Fig. 5:**
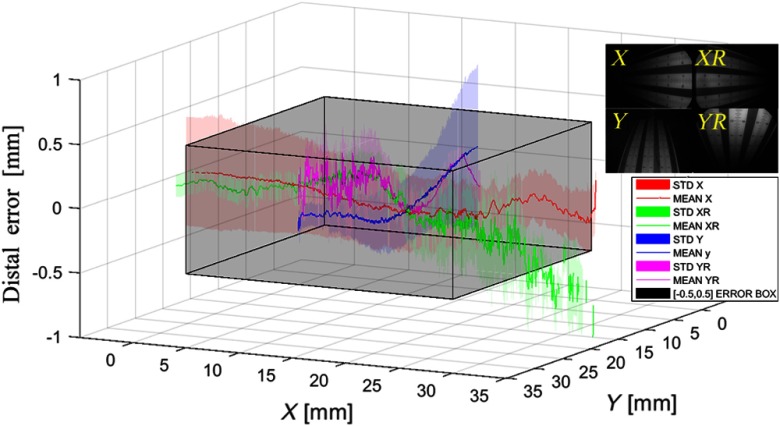
Precision of depth measurement with distal mean and standard deviation of point cloud distance to the fit plane in four directions as described in the inset along the x-axis (X in red color and XR in green color) and along the y-axis (Y in blue color and YR in magenta color). The gray boundary box indicates a distal error constraint between −0.5 and 0.5 mm.

**Fig. 6:**
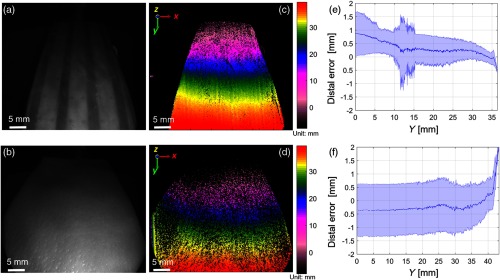
(a, b) Bright-field reflectance images of DOF target covered by intestine (top) and skin flaps (bottom). (c, d) The corresponding depth map of the tissue surface with depth color bar in millimeters. Camera exposure is set at 40 ms for each fringed image for the depth recording. (e, f) Precision of depth measurement with mean and standard deviation of distance from point cloud to the fit plane. Blue middle line indicates mean distance between 3-D point clouds to the fit plane, and the blue shade region indicates the standard deviation of the distal variations.

**Fig. 7:**
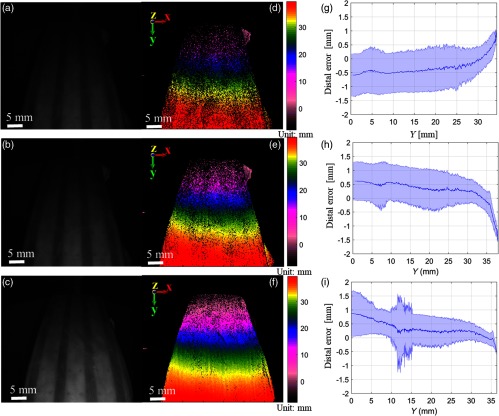
(a–c) Bright-field reflectance images of DOF target covered by intestine at different camera exposure times of 20 ms (top), 30 ms (middle), and 40 ms (bottom). (d–f) The corresponding depth map of intestinal surface with depth color bar in millimeters. (g–i) Precision of depth measurement with mean and standard deviation of distance from point cloud to the fit plane. Blue middle line shows mean distance, and the blue shade region marks the standard deviation of the distal variations.

To define the system sensitivity across the field of view, the 3-D depth error is computed with the DOF target in four rotational directions (Figs. 3–5). The 3-D depth error is defined as mean and standard deviation of all binning distances between the reconstructed 3-D point clouds to the fitted plane. Binning distances, selected with each binning width, are the ratio of the total data range along inclination direction [along the y-axis in Figs. 4, 6, and 7, for example] over the square root of the total number of data points. The fitted plane is calculated using the maximum likelihood estimation^39^ using MATLAB R2017b, Computer Vision System Toolbox™ with maximum allowed distance from the data inlier points to the fitted plane of 5  μm and maximum allowed absolute angular distance between the normal vector of fitted plane and the reference orientation of 3 deg. Due to the known planar geometry of the DOF target (45 deg), the reference orientation is computed by finding the principal direction (principal component) in which the majority of data points vary. To fulfill the sensitivity requirement for tissue imaging, the error calculation is also performed on DOF target with biological samples draped over the DOF target surface.

### 2.4 Sample Preparation

**Fig. 8:**
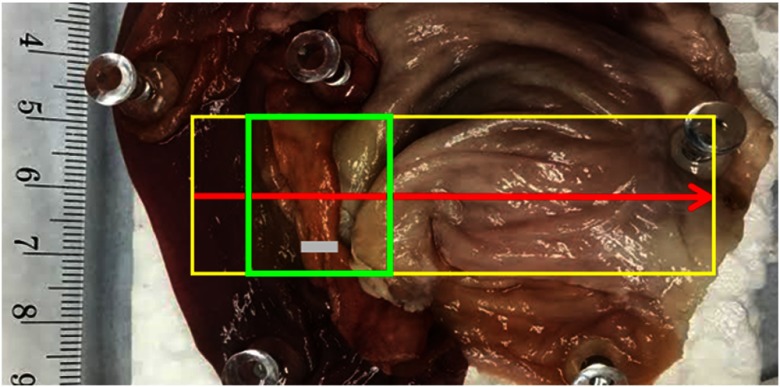
Digital color image of the intestinal sample for 3-D scanning in Fig. 9. The sample consists of smaller to larger intestines with different tissue pigments. The scanning area is within the yellow region, with scanning direction from left to right along the red arrow. Green region shows a demonstrated frame for 3-D depth still image excerpts from Fig. 9. Ruler unit is in millimeters.

**Fig. 9:**
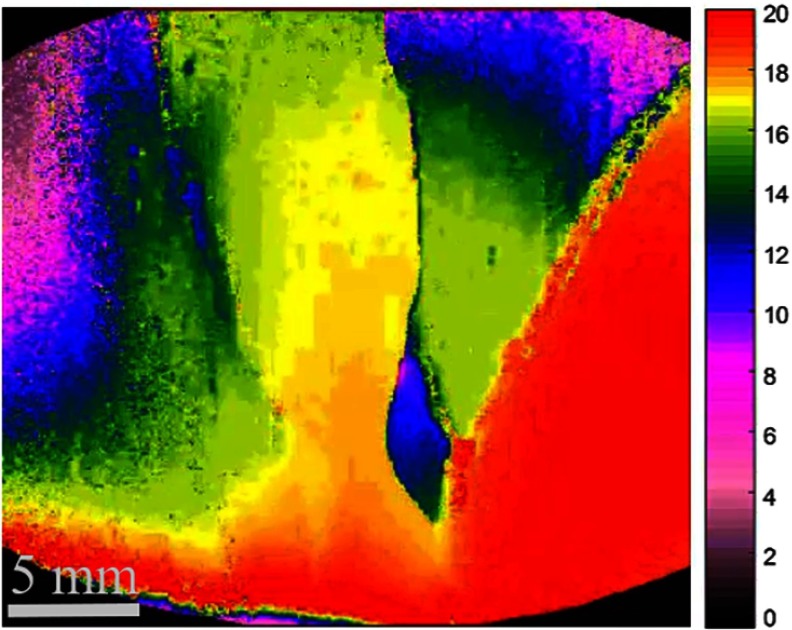
Still image excerpts from video recordings of depth map scanning (green ROI in Fig. 8) along the yellow region in Fig. 8. Camera exposure is set at 33 ms for each fringed image for the depth recording. The depth color bar is in millimeters. Video is set at 5× speed (Video 1, MP4, 1507 KB [URL: https://doi.org/10.1117/1.JBO.23.5.056009.1]).

**Fig. 10:**
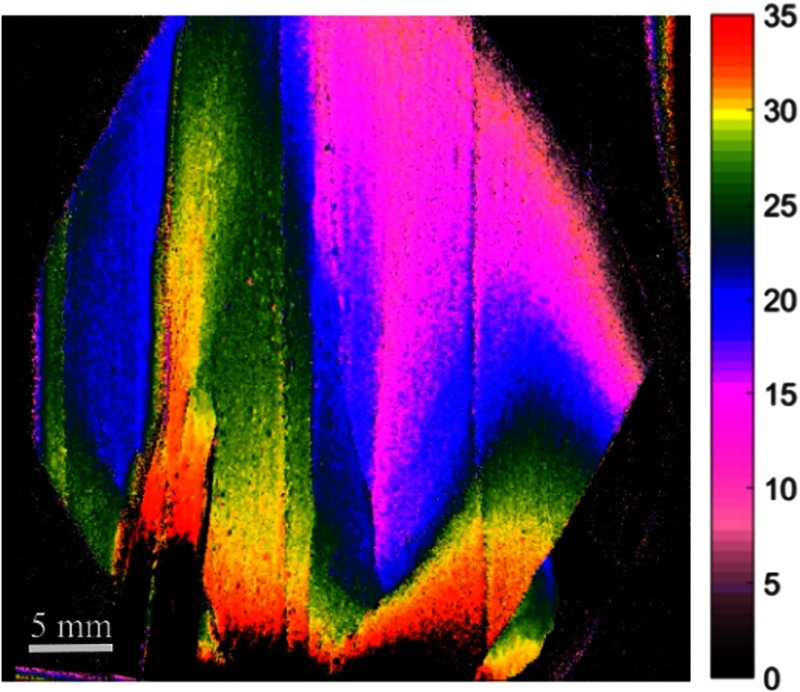
Still image excerpts from video recordings of depth map of anastomosis setup without looping suture. Forceps are used to show a simple typical tool manipulation during the 3-D recording. Camera exposure is set at 33 ms for each fringed image for the height recording. The depth color bar is in millimeters. Video is set at 5× speed (Video 2, MP4, 1254 KB [URL: https://doi.org/10.1117/1.JBO.23.5.056009.2]).

**Fig. 11:**
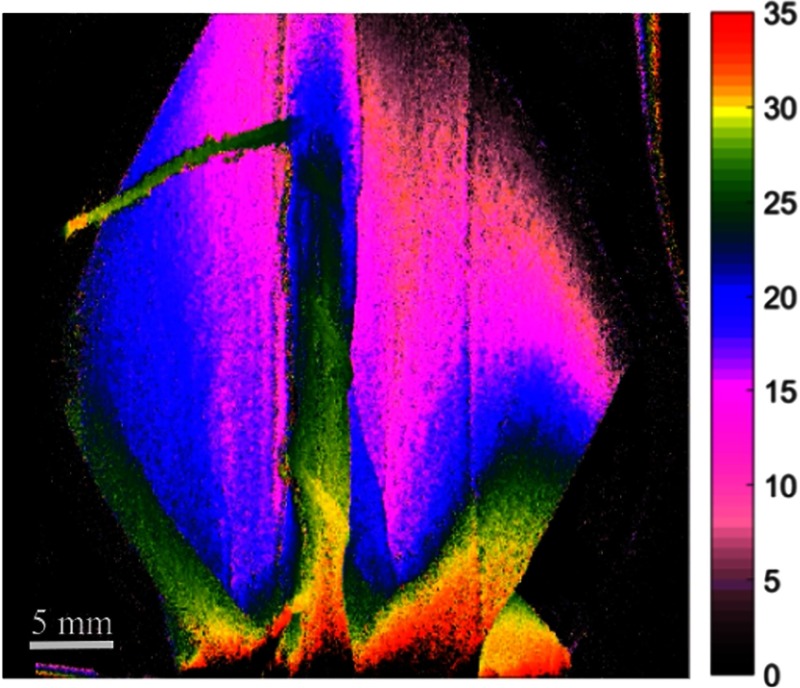
Still image excerpts from video recordings of depth map of anastomosis setup with looping suture lines. The depth color bar is in millimeters. Camera exposure is set at 33 ms for each fringed image for the height calculation. Video is set at 5× speed (Video 3, MP4, 660 KB [URL: https://doi.org/10.1117/1.JBO.23.5.056009.3]).

**Fig. 12:**
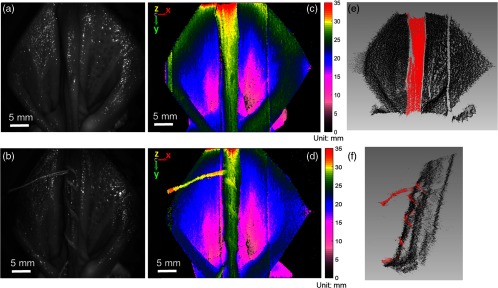
(a, b) Bright-field reflectance images of intestinal anastomosis at camera exposure time of 33 ms without (top row) and with looping sutures (bottom row) (top). (c, d) The corresponding depth map of intestinal surface with depth color bar in millimeters. (e, f) The corresponding 3-D point cloud distribution with segmented tissue fold and suturing stitches in red coded points.

The system sensitivity was evaluated using biological samples such as porcine skin and intestinal tissue. Porcine skin preserved with salt cure (Hormel Foods, Austin, Minnesota) was purchased from a local grocery. Samples of porcine skin samples were prepared by separating the skin and fat layers using a scalpel. The tissue samples were then rinsed using a pressure washer to ensure that the layer of porcine fat had been completely removed, and salt residue was dissolved. Samples of porcine intestine were purchased from a biological materials resource (Animal Technologies, Tyler, Texas). All intestines were prepared by washing biological waste from inside of the tissue, cutting the samples in 6″ lengths, and vacuum sealing the samples in freezer safe bags. Prepared samples of skin and intestine were then preserved in −20°C freezer (Sheikh Zayed Institute for Pediatric Surgical Innovation, Children’s National Health System, Washington, D.C.). Prior to imaging, the tissues were thawed to room temperature and used for the following tests:

1. Small intestine and skin samples were cut into flaps and placed onto the DOF target. 3-D images are collected to compute the depth error as mentioned in Sec. 2.3.
2. A mixture of small and big intestinal samples was piled and fixed on a Styrofoam platform inside a Petri dish. A video of 3-D measurements was recorded as the disk was translated (Figs. 8 and 9).
3. A setup using small intestine is used to replicate an end-to-end bowel anastomosis scenario. A porcine small intestine was transversally cut and four stay sutures were placed to expose the lumen opening in a diamond shape as shown in Figs. 10–12, where two sutures are closed to the mesenteric and antimesenteric corners of the lumen, and two additional sutures are in the middle, similar to a standard clinical staging technique.^40^ All stay sutures were suspended, and multiple running stitches were performed. A video of 3-D measurements was used to capture the depth profile of the intestines with the suture lines.

### 2.5 Data Acquisition

Several samples with different pigmentations are used in the system validation tests, such as the DOF target, small and large porcine intestines, and porcine skin sample. For each of the samples, a defined camera exposure time for capturing each fringed image is chosen for the best reflected photon counts and tissue feature contrast. In particular, 20-ms exposure time is set for DOF target, and 20- to 40-ms exposure time is set for other tissue samples. For each 3-D formation and point cloud display, a set of 16 fringed images are generated by the projector as an external trigger for each collected image by the CCD, creating in a closed-loop 3-D generating display with the speed of 16× exposure time.

## 3 Result

The 3-D depth map of a defined DOF target (45° inclement) is evaluated to validate the system sensitivity, with the coordinate origin denoting the reference zero plane (Fig. 3). Color bars displayed next to all the subsequent depth images and videos are used to refer the sample depth distribution within a defined range, and the reference zero plane is set as the farthest possible plane (from the distal end of the scope) that the scope can construct. Due to the depth perception, the farther object from the scope distal end appears to be smaller than with closer object. This only affects the depth map demonstration, not the point cloud calculation. The bar scale of 5 mm in all DOF depth map applies to the front of the sample. To minimize intensity-derived noise in depth calculation, only height measurements within the white regions on the DOF target [red ROIs in Fig. 3(a)] are selected. From Fig. 4, the three regions indicate similar depth errors within 0.25 mm in standard deviation (precision) and 0.008 mm in distal mean (accuracy) within the depth of field of 20 mm (relatively flat mean distal error from y=10  mm to y=30  mm). Regions outside the depth of field reflect either higher distal deviation or distal mean variation. For example, when the object is farther from the illumination (lower light distribution) from the origin of the coordinate system to y=10  mm, the distal deviation is as high as 0.8 mm; when the object is close to the illumination (causing specular light distribution), the distal mean varies up to 0.5 mm; notice that this mean variation occurs in left and right ROIs, i.e., when the fringes are distorted due to the scope parallax alignment.

To demonstrate the system sensitivity in its full field of view, we measure depth error of the DOF target in four directions along horizontal (X,XR) and vertical (Y,YR) axes as shown in Fig. 5. The distal error was measured within 0.5-mm standard deviation for the object within a $20\times 20\times 20\;{\mathrm{mm}}^{3}$ volume.

For tissue test 1, we use two tissue samples of small intestine (mean thickness of 0.497 mm measured by caliper ruler) and skin (mean thickness of 3.490 mm measured by caliper ruler) as shown in Fig. 6. Each fringed image of both samples was imaged at the same camera exposure of 40 ms. The depth maps indicate the designed inclination of the DOF target, as well as features on the tissue such as more homogeneous texture on the skin compared with inhomogeneous texture in intestine. Moreover, the thinner intestine tissue results in a depth noise of 0.5-mm standard deviation, which is lower than in the case of thicker skin tissue, with standard deviation of about 1 mm for the depth noise. The noise magnitude increase in the case of the intestine between 10 and 15 mm in Fig. 6(e) is due to the excess tissue protrusion at the right edge of the target. When the thickness is small (tissue is almost transparent), the average distal error is less than in the thicker tissue due to the more sharply defined vertical fringes. The higher contrast of the vertical fringes leads to this increased sharpness and increase in the sensitivity of the depth reconstruction. With samples such as porcine skin, the surface is rather flat on top of the DOF with dimension of $35\times 50\;{\mathrm{mm}}^{2}$. In future work, we will focus on the comparison of this laparoscopic approach with other commercial free-space and endoscopic 3-D reconstruction methods.

To improve lighting conditions in tissue imaging, either LED power or exposure time of the camera sensor can be increased. In our case, we chose to increase the camera exposure due to the LED power and the scope transmission limit. With camera exposure time increments at 20, 30, and 40 ms, more data points are collected; therefore, the noise variation in the case of intestinal 3-D is reduced from around 0.8 to 0.5 mm (Fig. 7).

In tissue test 2, to confirm the performance of the system on biological tissue in video mode, we recorded 3-D measurements of a mixture of porcine intestines on a translated platform to mimic a realistic anastomosis environment. For videos of all intestinal samples, the camera shutter is set to be around 30 ms (camera received frame rate of 32 fps) to collect the fringed image. For each group of 16 fringed frames, a depth calculation is recorded, resulting the speed of the 3-D collection to be at 1.57 fps. The tissue consisted of a variety of thickness and tissue pigments, which were detected by the system. The sample ROI in Fig. 8 (green ROI) is scanned along the red arrow and within the yellow boundary. The depth map of the scanned ROI is shown in Fig. 9.

We next conducted an *ex vivo* experiment with the end-to-end anastomosis in porcine bowel tissue (tissue test 3). The experiment evaluates the detection of suture, tissue deformity, and the surgical tools at a reasonable speed in a clinically relevant setting. The system performed this detection dynamically and quantitatively in metric colormaps as displayed in the video demonstration with surgical tool and looping suture in Figs. 10 and 11. Similar to previous depth images, a depth scale from 0 to 35 mm is used to display depth profile from the zero-reference plane to the interested sample height; therefore, when the surgical tool is presented, part of the tool is closer to the distal end of the scope, out of the studied range and therefore was not displayed. The reconstruction records a dense point cloud of the tissue surface without holes that are often collected in homogeneous surfaces. In case of no suture line in Figs. 12(a), 12(c), and 12(e), the middle tissue section detects flap features marked in red. In Figs. 12(b), 12(d), and 12(f), the three suture lines are segmented based on intensity thresholding and marked in red in Fig. 12(f) with an average height of 0.726±0.0075  mm above the baseline compared with a real height of suture line measured by caliper of 0.723±0.022  mm.

## 4 Discussion

Using SI technique, the system achieves a dense 3-D constructed point cloud of tissue sample. Compared with techniques such as ToF and plenoptics imaging, the system gives a higher quantified depth sensitivity in pigmented sample and the flexibility by adapting the design in an endoscopic setting with different focusing mechanism using other focusing lens and fringe design. In addition, the system provided adequate point cloud distribution for robotic navigation, with no or little data “holes” as in stereoscopy or other hybrid laser dot-like structure illumination. The propose system is a compact design using a low-power illumination source; hence, mounting the system to mechanically controlled with another surgical robotic arm is feasible. In future work, we focus on the development of this harmonic motion for use in multiple surgical access points.

Analyzing the current hardware, the proposed system speed of 1.57 fps is mainly due to camera sensitivity when imaging tissue samples and the scope transmission. The scope transmission is measured to be around 40% using a collimated laser diode at 532 nm. With the LED illumination built in the projector optical chamber, this transmission is restricted to around 7% due to the high angle dispersion (the measured power at the distal end of the scope is around 0.8 mW, compared with 12 mW at the proximal end of the endoscope). For a faster speed, several improvements can be achieved. First, a higher light delivery for both illumination and imaging scopes can be used, either by increasing LED power, or increase the scope diameter. By replacing the current scopes with two identical 1-cm scopes, the 3-D speed is measured to be about around 7 fps, with 10 ms for each fringed image under the same system hardware and image resolution.

The residue fringes and motion artifacts are observed in both the videos, and this issue is directly related to the speed of our imaging system. Fortunately, the dominant tissue motion that we encounter for the intestine procedures is a slow and periodic motion related to patient breathing. Thus, for the relatively low imaging speed of 1.57 fps, as seen in the videos, it does not pose a significant issue. However, it does introduce relative position error for faster motion and can only be corrected if we improve the speed of our imaging system. The improvement of the system speed will be the subject of our future work.

From the demonstrated 3-D, the projected fringes provide enough structural information to reconstruct homogeneous surfaces of the intestines without the use of additional markers. With the baseline between the imaging and illumination scopes of 12 deg, the system requires a dual access port or a single access port of about 12-mm diameter for the two 4-mm scopes into a 40-mm insertion depth. Future design of the system aims to reduce the port diameter using smaller and more flexible endoscope.

## 5 Conclusion

This paper reports the design, assembly, and testing of an endoscopic SI imaging system for visualization and guiding of intestinal anastomosis. A systematic study was performed to assess the system accuracy for the image guided surgery. The system provides a quantitative depth information on static objects such as the known DOF target with an accuracy of 0.008 mm and precision of 0.25 mm within a depth of field of 20 mm, as well as depth reconstruction of dynamic intestinal structure in anastomosis situation. The demonstration of 3-D reconstruction videos confirms the feasibility of this technology in a clinical setting for anastomosis surgery in endoscopic setting.
